# Design of a Chipless RFID Tag to Monitor the Performance of Organic Coatings on Architectural Cladding

**DOI:** 10.3390/s22093312

**Published:** 2022-04-26

**Authors:** Tim Savill, Eifion Jewell

**Affiliations:** Materials Research Center, College of Engineering, Swansea University Bay Campus, Swansea SA1 8EN, UK; e.jewell@swansea.ac.uk

**Keywords:** chipless RFID, corrosion, organic coating, degradation, sensors

## Abstract

Coating degradation is a critical issue when steel surfaces are subject to weathering. This paper presents a chipless, passive antenna tag, which can be applied onto organically coated steel. Simulations indicated that changes associated with organic coating degradation, such as the formation of defects and electrolyte uptake, produced changes in the backscattered radar cross section tag response. This may be used to determine the condition of the organic coating. Simulating multiple aging effects simultaneously produced a linear reduction in tag resonant frequency, suggesting coating monitoring and lifetime estimation may be possible via this method. For coatings thinner than calculations would suggest to be optimum, it was found that the simulated response could be improved by the use of a thin substrate between the coated sample and the antenna without vastly affecting results. Experimental results showed that changes to the dielectric properties of the coating through both the uptake of water and chemical degradation were detected through changes in the resonant frequency.

## 1. Introduction

Organic coatings are widely used in the construction industry for aesthetic, corrosion protection, and weather resistance reasons. In fact, in 2020, the EU produced 130 million metric tonnes of hot rolled steel, of which 5 million metric tonnes was organically coated [1]. Despite this, it has been suggested by some studies that the reason for the high cost of corrosion is due, in part, to the poor selection of protective measures [2]. Hence, one widely suggested strategy for corrosion protection is to ‘develop advanced life prediction and performance assessment methods and to move to a greater degree of corrosion monitoring [3].

Currently, coatings used on construction panels are rarely monitored in-situ and the expected performance of the overall building envelope is often only estimated from lab-based accelerated corrosion testing. Real time monitoring could allow more accurate estimates of building cladding lifespan, as well as required maintenance schedules, providing the customer with active performance data [3]. A significant amount of emerging research in this field shows the appetite for this technology [4].

An ideal solution would be a wireless, internet of things style sensor system which allows remote, live monitoring of the organic coatings. The oil and gas industry have long been aware of the benefits of corrosion monitoring of pipelines [5]. However, they are currently the only significant industry with commonly used, commercially viable monitoring methods. The construction industry is starting to catch up, although current research suggests monitoring is focused on load critical components, especially reinforced concrete rebar. 

Some research has been carried out into coated panel sensors, such as strain gauges [6], corrosion indicating paint [7], electrical resistance probes [8], and numerous EIS (electrical impedance spectroscopy) modifications [9,10,11,12,13,14,15,16,17]. These sensors work in small scale testing. However, they suffer from the same drawbacks of practical use. For example, they often either modify the paint system, require connection to the underlying metal substrate, or require modification between the layers of the coating. This makes these systems difficult to implement and replace if necessary, and, in some instances, reduce the effectiveness of the coating itself.

Sensors for monitoring coated steel products have to be capable of detecting the kind of changes in coatings that are indicative of degradation or failure. These include the formation of defects and the associated uptake of oxygen and ions from the environment, coating adhesion loss, electrolyte penetration, or corrosion initiation [18]. Furthermore, degradation can also produce increased porosity of the coating through the action of UV, blistering due to osmotic or electrochemical effects, and decreased coating thickness through erosion and coating mineralization and oxidation [18,19,20,21]. Tracking the spread and size of corrosion effects would also be required to allow an indication of the severity and overall condition of the product. Cut edge corrosion, when corrosion begins and propagates from a metal edge exposed during manufacture, is one of the most commonly seen defects for organically coated steel [22].

This paper outlines the basics of a new passive RFID (resonant frequency identification) technique that aims to allow easy performance monitoring of organically coated steel cladding products without some of these drawbacks. A passive RFID sensor presents a number of promising features for organic coating monitoring. As a cheap and ‘semi-remote’ method, it would allow for a medium range, non-destructive monitoring method of a number of assets. In fact, a significant amount of work has been carried out on developing RFID based sensors [23,24,25]. The approach taken by the vast majority of the sensors currently being developed is to use a so called ‘chipless’ RFID design.

Chipless RFID tags were first considered as a concept in order to allow RFID tagging to compete financially with low-cost barcode tagging [25,26]. By removing the integrated circuit, chipless RFID is greatly reduced in cost and allows consideration of fully printable tags, increasing ease and rate of production [26]. Chipless sensors offer a much simpler approach in which some of the complications of impedance matching as well as other complications are negated compared to chipped devices. 

**Figure 1:**
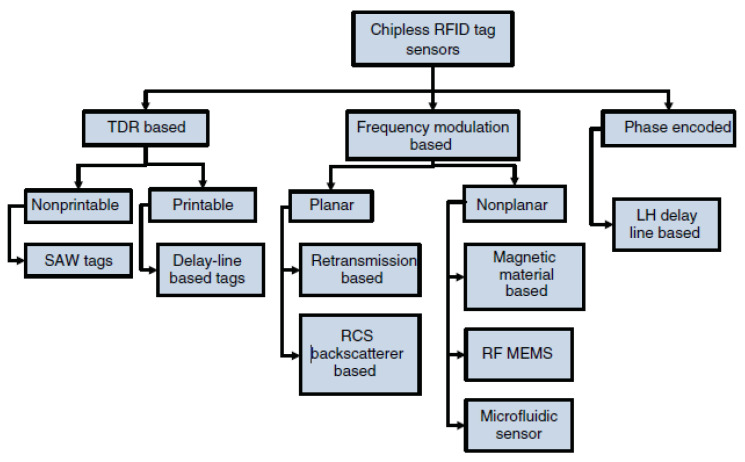
Types of chipless RFID tags (Reprinted with permission from Ref. [25] 2016, John Wiley and Sons).

As a sensor, they are stated to have better robustness, lower radiated power, and longer life than traditional sensors [25]. Three types of chipless RFID sensors exist, namely time-domain reflectometry (TDR), frequency modulation, and phase encoded sensors [25], and these are summarized in Figure 1. For each of these types, different tag technologies exist, such as surface acoustic wave (SAW) and radar cross section backscatter (RCS).

**Figure 2:**
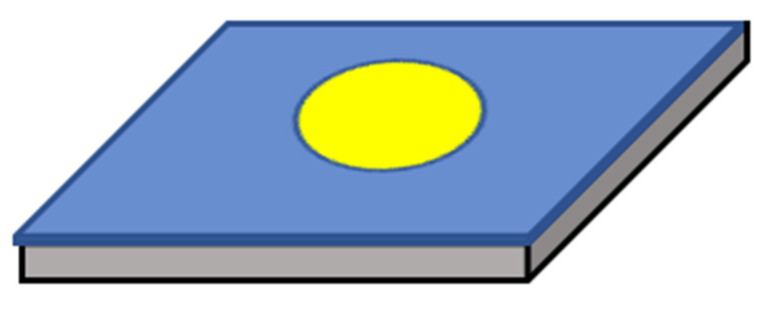
A basic CMPA.

A recent study involved using a chipless RFID circular microstrip patch antenna (CMPA) to monitor crack growth in aluminium [27]. Microstrip antennas are commonly used in wireless communication, including in high performance situations, such as in satellites and aircraft [28]. They have advantages of low price and profile, simplicity, and versatility [28]. A basic microstrip antenna is composed of a ground plane, which is covered by a dielectric layer with a metal antenna strip on it. A microstrip patch antenna is differentiated because it consists of relatively large sections, or patches, of metal [29]. A CMPA is simply an MPA in which the patch is circular in shape, as shown in Figure 2.

**Figure 3:**
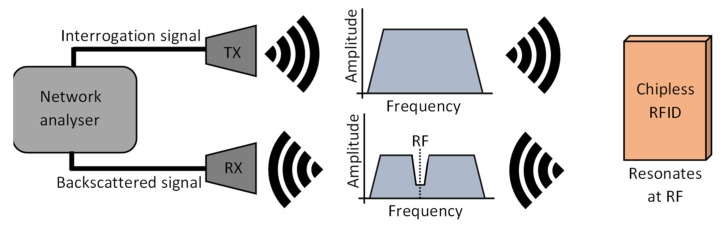
Basic working principle of an RCS backscattered RFID tag.

In this paper, a similar approach was used to that in [28], which utilizes radar cross section (RCS) backscatter-based technology, which is based on frequency modulation. The principle is described visually in Figure 3 and involves the use of a microstrip resonator. An interrogation signal is aimed at the chipless tag which will resonate at a unique frequency and hence produce a signature backscattered signal which is received by a further antenna for analysis [25,27].

The monitoring system developed in this paper has parallels to that designed by Marindra [27], which utilised the fact that the resonant frequency of a CMP antenna is affected by changes in the ground plane. This study aimed to use a similar antenna system to monitor changes in the dielectric layer of such a system with the dielectric layer, in this case, being an organic coating layer on a steel product.

As described in [28], the dominant TM^z^_mn0_ mode for a circular microstrip patch antenna is the TM_110_ mode for which the resonant frequency is defined by:

(1) $${\left(f_{r}\right)}_{110}=\frac{1.8412}{2\pi a_{e}\sqrt{\mu \epsilon }}=\frac{1.8412v_{0}}{2\pi a_{e}\sqrt{\epsilon_{r}}}$$

where:

(2) $$a_{e}=a{\left\{1+\frac{2h}{\pi a\epsilon_{r}}\left[ln\left(\frac{\pi a}{2h}\right)+1.7726\right]\right\}}^{\frac{1}{2}}\;$$

where a is the diameter of the circular patch, h is the thickness of the dielectric layer, $\epsilon_{r}$ is the relative permittivity of the dielectric layer, $v_{0}$ is the speed of light, μ is the permeability of the dielectric, and ϵ is the permittivity of the dielectric layer.

Hence, it can be concluded that:

(3) $${\left(f_{r}\right)}_{110}\propto \;f\left(\epsilon_{r}\right)\;$$

(4) $${\left(f_{r}\right)}_{110}\propto \;f\left(h\right)$$

Therefore, the resonant frequency of a patch antenna is related to a function of both the relative permittivity (dielectric constant) and the height of the dielectric. As a result, any changes to the dielectric properties of an organically coated product could be detected via this approach. This would include degradation due to water ingress, defect formation, or paint formation failures such as chalking. This approach was utilized by [28] to monitor the response of an epoxy coated resonator and was able to detect changes in the dielectric properties of the epoxy coating produced by the absorption and desorption of water. The proposed method differs from this method as the antenna can be placed onto, rather than under, the coating of interest which may interfere with the adhesion and/or protective properties of the coating. Furthermore, as the proposed device is solely reliant on backscatter RCS measurement, a physical wired connection to the antenna, as in [30], is not required simplifying the procedure.

The aim of the investigation was to develop the concept outlined, in order to prove its applicability to architecturally painted steel. The strategy employed used a combination of simulation and laboratory experimental techniques. The simulation provided a means of estimating the response change when variations in material properties which could be expected during degradation occurred within a purely simulation space. The simulated response could then be compared and validated using the laboratory derived experimental results. This parallel approach ensured that a greater understanding of underlying physics could be established while also proving the applicability in the real world.

## 2. Methodology

### 2.1. Simulation

**Figure 4:**
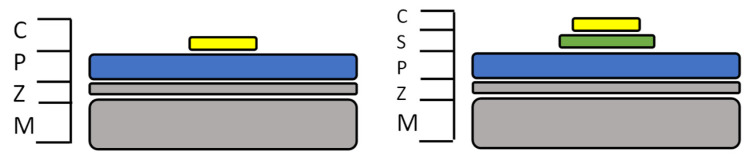
Two tag systems NS, no substrate, (**left**) and S, substrate, (**right**) showing the copper sensor (C), the substrate layer (S), the paint system (P) the zinc galvanized layer (Z) and the steel metal substrate layer (M).

The tag was designed and tested in CST (Computer Simulation Technology) Design Environment 2019, a 3D electromagnetic analysis software commonly used for antenna design and analysis [31]. Two approaches, the basic design of each shown in Figure 4, were considered. The first approach (NS) solely used the organic coating as the dielectric layer, and the sensor patch is attached to this, while the second approach (S) used a dielectric substrate between the sensor patch and the coated steel panel.

**Figure 5:**
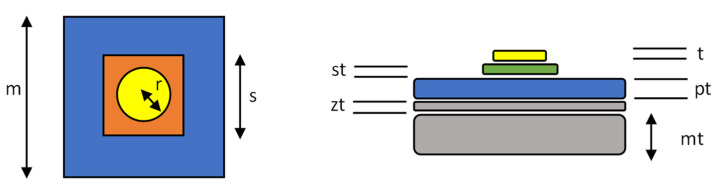
Tag systems showing key dimensions which are defined in Table 1.

Two coating systems were also considered and designed in the software based on two commonly used coated steel products composed of a steel substrate, a zinc metal coating layer, and a dielectric organic coating layer. This is referred to as coated steel in the following work. A polyvinyl chloride (PVC)-based coating with a total nominal coating thickness of 211 microns and a polyurethane (PU)-based coating with a total nominal coating thickness of 41 microns were designed. These are two commonly used coating systems for architectural steel cladding and are referred to in the following work as PVC and PU. The dimensions of the tag are displayed in Figure 5 and listed in Table 1.

In the modelling of the cladding panels, several assumptions were made for the initial testing. Firstly, the coating layers of pre-treatment, primer, and topcoats were combined into one homogenous layer with a cumulative thickness and an arbitrary dielectric constant of 3.5. The metal substrate was defined as 1010 steel and the metallic coating layer as pure zinc. The size of the cladding piece was set to 6 cm^2^, and the substrate layer used was FR-4 (lossy). The properties for each of the materials used are shown in Table 2.

Although the actual steel substrate and zinc layer are composed of either different or more complex alloys, the conductivity values used were expected to be a fair representative value. Furthermore, additional simulations, not provided in this work, showed that small deviations in these values of conductivity did not noticeably affect the resonant frequency and only produced small changes in the measured RCS. 

Perhaps the largest source of uncertainty is the accuracy in modelling the coating layers as one homogenous layer, whereas in reality the pre-treatment, primer, and topcoat(s) have different requirements and hence compositions. This was mainly done for ease of modelling, and it is believed to be representative of a weighted average of the multiple dielectric layers. It is possible that experimental confirmation of this assumption may be further required. A dielectric constant of 3.5 was considered an accurate representation of both systems as the coatings are based on polyvinyl chloride chemistry, which has a stated dielectric constant of 3.4 [34,35], and polyurethane chemistry, which has a stated dielectric constant of 3.2–3.6 [36,37]. 

**Figure 6:**
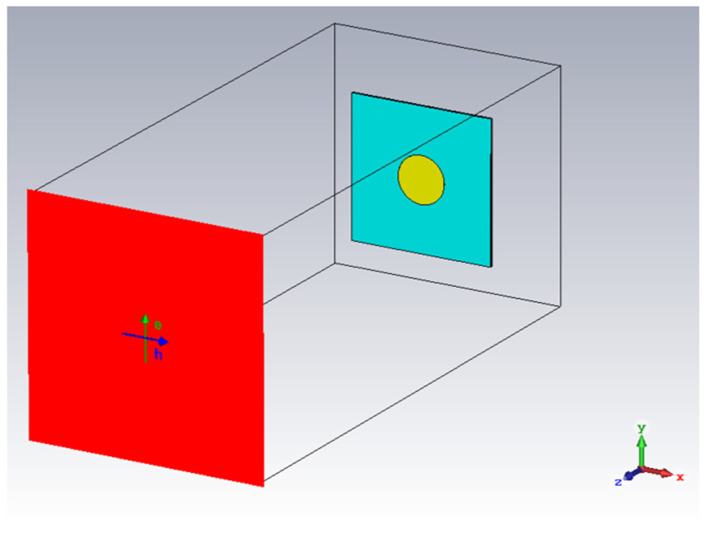
Simulation set up in CST Studio Suite.

The simulation, shown in Figure 6, was set up with a frequency range of 2–6 GHz of plane wave excitation in the z direction. The E-plane of the excitation plane wave was oriented parallel with the y axis and the H-plane was oriented parallel with the x axis. The plane wave excitation source and an RCS Probe were set −200 mm away from the sample in z direction to provide the simulated interrogation signal and response signal measurement of the tag, respectively, at 200 mm distance. Finally, all boundaries were set to open (add space), which is used to simulate free space surrounding the set up and is recommended for antenna calculations [38].

The resonant frequency of the tag was determined by measuring the frequency at the point of maximum drop of RCS amplitude. The RCS change at resonant frequency was calculated by taking the measured RCS (RCS_RF_) away from the average of the RCS at the beginning of the valley (RCS_L_) and the RCS at the end of the valley (RCS_H_):

(5) $$\Delta \mathrm{RCS}=\frac{{\mathrm{RCS}}_{L}+{\mathrm{RCS}}_{H}}{2}-{\mathrm{RCS}}_{\mathrm{RF}}$$

### 2.2. Production of the Antenna

**Figure 7:**
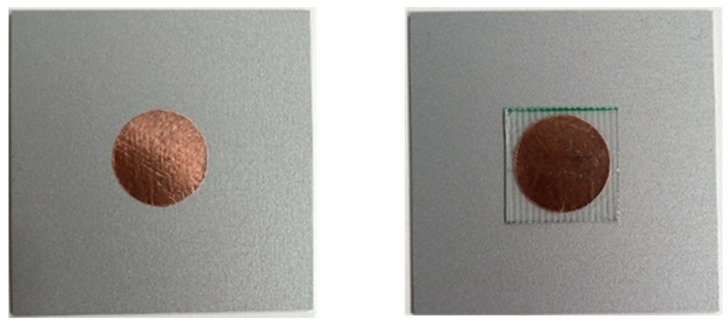
The manufactured Chipless RFID Tag deployed onto the same without (**left**) and with (**right**) the substrate layer.

**Figure 8:**
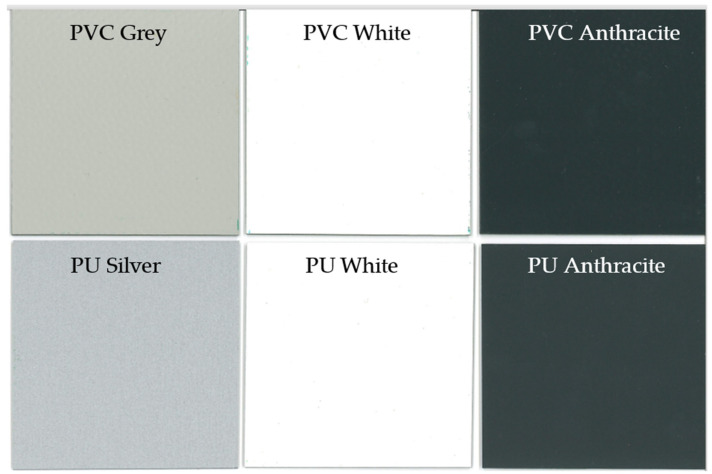
The samples used in this study.

In order to validate the simulations, several tags were produced, as shown in Figure 7. The antennae were manufactured from adhesive copper tape (RS Pro foil, thickness 0.035 mm, from RS Components, Ireland) using a 20 mm paper punch. This method was initially used for simplicity and because it gave reasonable cut quality and uniformity. As copper rapidly tarnishes antennae were also produced from adhesive aluminium tape (RS Pro foil thickness 0.04 mm), this is a far more environmentally resilient substrate and hence it was considered as an alternative. The 20-mm circles were then either attached directly to the sample, or to the Fr-4 substrate, which was attached to the sample via thin double sided adhesive tape. An example of the two produced antenna systems is shown in Figure 7. A number of coated steel samples were used in this study with the colours used including white, anthracite, grey (PVC only), and silver (PU only). This was done to understand the effect the different coating colours may or may not have on the measured resonant frequencies. The samples used are shown in Figure 8.

### 2.3. Antenna Measurement

**Figure 9:**
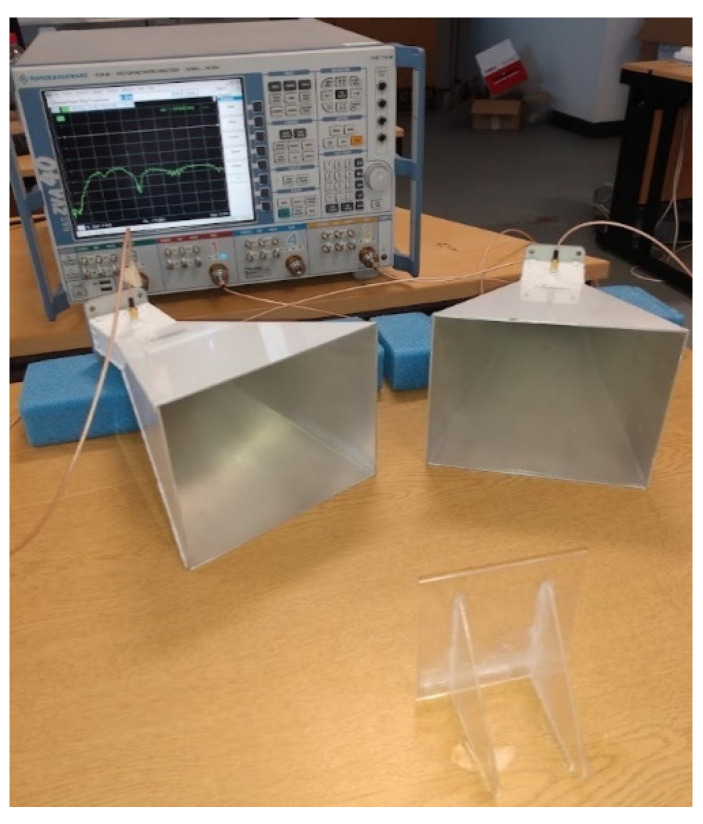
VNA and horn antennas used to interrogate the sample.

To measure the antenna response, a vector network analyser (VNA) (model) was used, connected to two horn antennas (TX and RX) (model). A signal sweep from 4 to 6 GHz was emitted from the TX horn antenna and the resulting S_21_ from the RX antenna was recorded. To ensure similar positioning of the antenna in each test, a small acrylic stand was produced, and the position of each horn antenna was marked on the test workspace to ensure the same range was used. The distance between horn antennas was 5 cm and the distance to the sample was 20 cm as with the simulations. The test set up is shown in Figure 9.

## 3. Results

### 3.1. Simulations

#### 3.1.1. Initial Simulations

**Figure 10:**
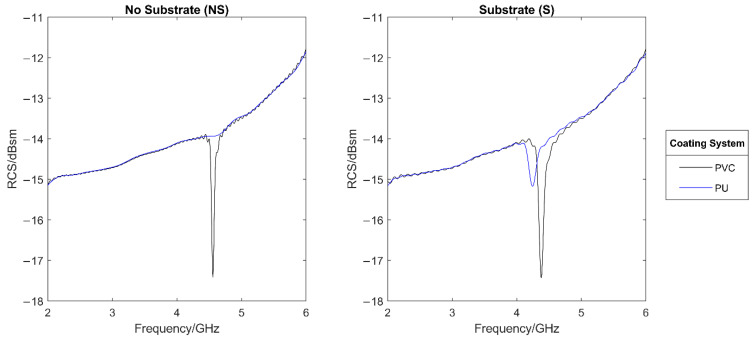
Simulated RCS response of sensors mounted directly on coated system (NS, **left**) and sensors mounted on an additional substrate (S, **right**).

The simulated RCS response for each system is shown in Figure 10 and the main results that can be drawn from each RCS response are summarized in Table 3. It can be seen that the PVC system gives a similar response when the antenna is placed both directly on the coated steel and on a substrate. The PU coating system, comparatively, does not show a very significant response when the antenna is placed directly on the cladding. However, a much more prominent, albeit smaller than the PVC system, response is recorded when a substrate layer is used. Both systems show a small shift to a smaller resonant frequency when the substrate layer is used. This is expected as the substrate has a larger dielectric constant of 4.3. Adding this larger value dielectric layer has the effect of decreasing the resonant frequency of the system according to Equation (1).

As the only difference in simulations between system PVC-NS and PU-NS is the paint thickness, the poor response from the PU system (PU-NS) must be caused by the change in dielectric layer thickness. It is stated in [28] that the usual dielectric substrate height h for a microstrip patch antenna with a dielectric constant between approximately 2 and 12 is:

(6) $$0.003\lambda_{0}\leq h\leq 0.05\lambda_{0}$$

The calculated corresponding height range for the observed resonant frequencies seen is shown in Table 4. It can be concluded that the optimum range is not satisfied by the PU system paint thickness unless it is further increased with the substrate used in PU-S. As stated in [39], a decreased thickness of dielectric can lead to greater losses and a decreased efficiency, thus explaining the lack of a significant response by the PU model.

**Figure 11:**
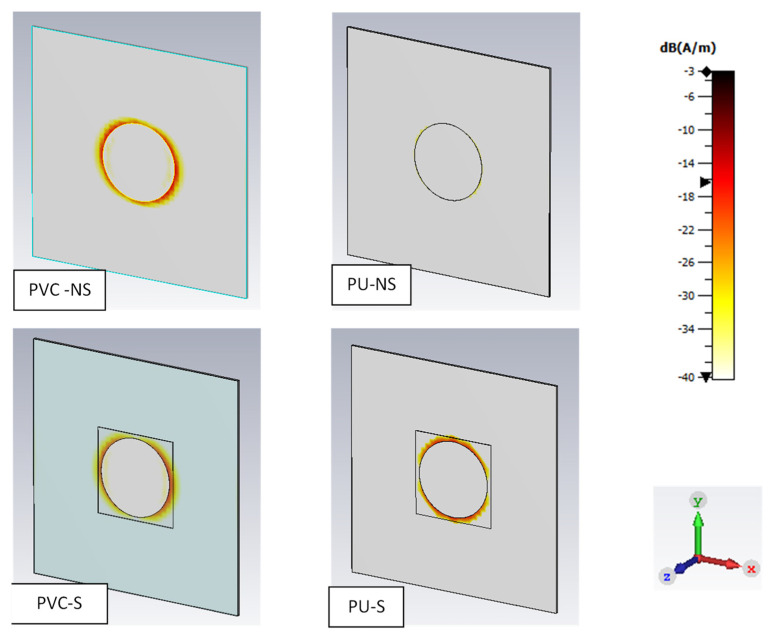
Simulated surface current distribution on each system at resonant frequency.

This conclusion is reinforced by comparing the surface current distribution maps of each system at resonant frequency, as shown in Figure 11. It can be seen that the PU sample with no substrate has a significantly lower current distribution surrounding the antenna patch than any of the other systems. This is thought to be down to the poor efficiency of the antenna because of the thin dielectric layer, which causes far less resonance and hence electrical current. As a result, the backscattered RCS records far less of a change in the signal as little power has been transferred.

#### 3.1.2. Simulated Changes in Dielectric Constant

To monitor the effect of changes in the dielectric constant of the paint layer, a parametric sweep was performed in which the dielectric constant of the paint was changed from 2 to 5 in increments of 0.2. 

**Figure 12:**
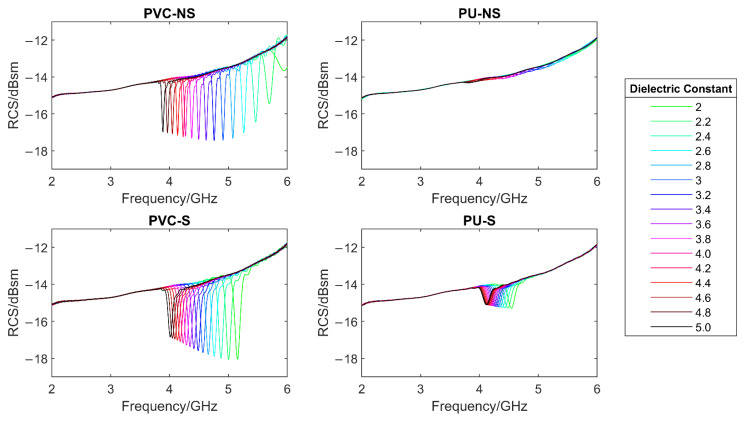
The effect of changes in the dielectric constant of the paint layer on the simulated RCS response.

**Figure 13:**
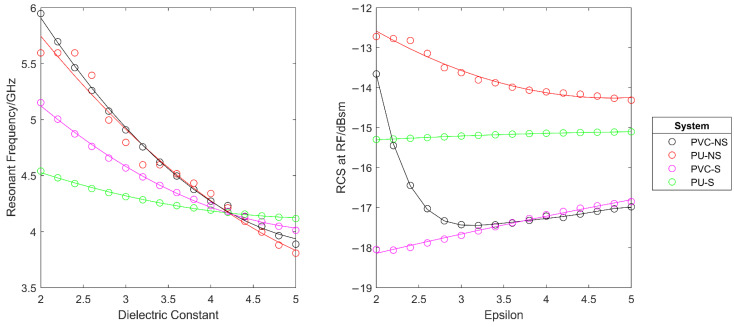
The effect of changes in the dielectric constant of the paint layer on the resonant frequency (**left**) and the RCS at resonant frequency (**right**) of each system.

As can be seen in Figure 12, for all systems, a clear trend was seen in that as the dielectric constant was increased, the resonant frequency of the system decreased. This is expected from Equations (1) and (3). However, it was also seen that the use of the substrate layer decreased the magnitude of the resonant frequency change. In Figure 13, it can be seen that the total change in resonant frequency for systems PVC-NS and PU-NS is far greater than that for PVC-S and PU-S. This is because the substrate has an unchanging dielectric constant and so is thought to average out some of the dielectric changes introduced. 

What is perhaps more unexpected is that, by using a substrate layer, the RCS at resonant frequency showed a far clearer trend of reducing with increased value for the dielectric constant. This can be seen by comparing the four systems against each other, as shown in Figure 13. 

This set of results helps to validate the model from the assumption that a dielectric constant of 3.5 was used and hence provides more confidence in the simulation process. If the coating system used has a different dielectric constant, a result will still be expected, however it will show a shift to a lower frequency, as shown by this simulation.

#### 3.1.3. Simulated Aging/Degradation

In reality, as a coated steel sample ages, a number of the effects simulated above occur simultaneously. Hence, it is important to determine the effect of multiple expected changes on the response. It is also important to determine if the multiple effects cause the overall change in response to reduce or even cancel out. To do this, a test was performed in which aging and degradation of the coating was simulated eight times and with each iteration. As displayed in Table 5:

- The dielectric constant of the paint layer was increased by 0.025 to simulate water ingress. Previous work such as [30] has shown that water uptake by a coating leads to an increased dielectric constant of up to at least 8%.
- The diameter of small defect holes in the paint were increased by 0.01 mm to simulate defect growth. These were designed to be placed ‘randomly’ with no specific pattern to attempt to mimic as close as possible that which would occur in reality. The defects were placed centered on (−4, 0) (3, 3) (−3, −5) (−2, 7), with (0, 0) being the centre of the CMPA. This sort of defect is a known failure method in organic coatings as a result of mechanical shock and/or aging [40].
- The paint thickness was decreased by 0.001 mm to simulated UV degradation. A decrease in organic coating thickness is known to occur through chain scission as a result of organic coating exposure to UV, oxygen, and other atmospheric contaminants [41].

**Figure 14:**
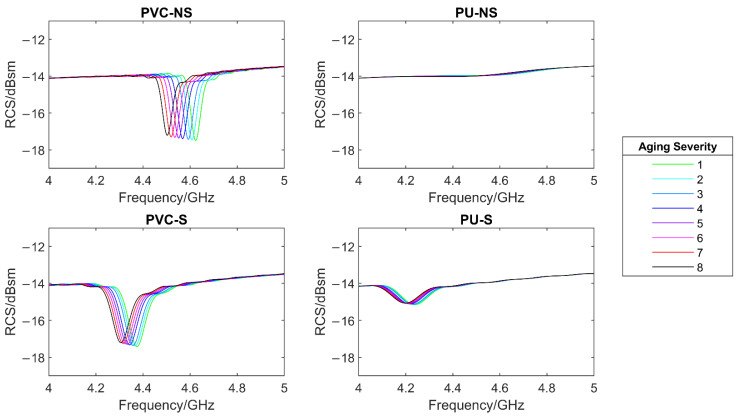
The effect of simulated aging on the simulated RCS response of each system.

**Figure 15:**
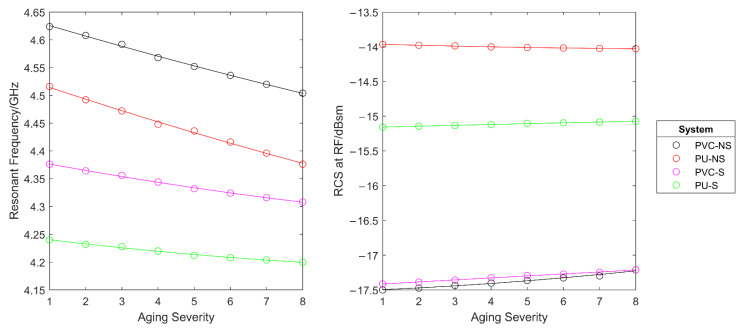
The effect of simulated aging on the resonant frequency (**left**) and the RCS at resonant frequency (**right**) of each system.

This offered a reasonable level of replication of the real-life ageing process of a coated specimen which undergoes the changes simulated in this exercise. The simulated RCS response for each system is shown in Figure 14 and the effect of the aging severity on the resonant frequency and the RCS at resonant frequency are shown in Figure 15. It can be seen that the resonant frequency shifts to lower frequencies as the simulated aging severity increases. Furthermore, the RCS at resonant frequency is seen to decrease in magnitude with simulated aging. Using a substrate had little effect on the change in frequency with aging. However, as before, this makes identification of resonant frequencies in the thinner paint system far easier, by increasing the depth of the trough.

### 3.2. Experimental

#### 3.2.1. Initial Test of the System

**Figure 16:**
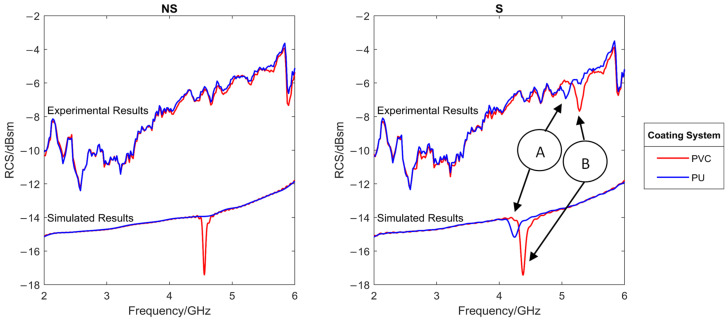
Comparison of the simulated and measured RCS response of sensors mounted directly on coated system (NS, **right**) and sensors mounted on an additional substrate (S, **left**). Identifiable peaks are matched between simulated and experimental results with designators A and B.

Figure 16 shows the comparison between the simulated and measured RCS for both paint and mounting systems. This test was carried out on virgin, white coloured samples. It can be seen that interference was captured compared to the ideal simulated scenario. This made the detection of the resonant frequencies for the NS samples (no additional substrate) impossible to determine. However, the resonant frequencies of system two (marked A and B) were detectable. These were observed to be at a higher frequency and provided a smaller RCS drop that was predicted by the simulations. However, this is not unexpected due small variations between the values and assumptions used in the simulations and the actual real values as mentioned previously in Section 3.1. The resonant frequency troughs did however appear in a similar position and size relative to each other when compared to the simulated results giving confidence in their correct identification. While it is true that noise would be greatly reduced through the use of an anechoic chamber, this would somewhat defeat the desire to use this technique in the field. While, due to the low simulated RCS trough, it was expected that the NS-PU sample was undetectable, it was surprising that the NS-PVC sample also produced no clear resonant frequency trough, especially as in simulations this showed a clear decrease in RCS of around 3.5 dBsm at resonant frequency. It was theorized that this discrepancy between simulation and experimental may be due to the difference between modelling the coating perfectly as a layer of set dielectric properties and measuring on what is an inhomogeneous, multi-layer, multi component, complex paint system. While in a simulation, the paint system alone can produce a resonant antenna, in real life, the addition of a small FR-4 substrate with homogenous dielectric properties is required to support the resonance of the antenna. Hence, it was concluded that the concept of attaching the circle directly to the paint without an additional substrate was insufficiently responsive to use for further testing.

However, to reduce the impact of noise, the following results were calculated by subtracting the sample background RCS. This was the measured RCS of the sample without the antenna attached. As shown later, this allowed far easier trough detection and noise removal.

#### 3.2.2. Detection of Artificial Weathering

**Figure 17:**
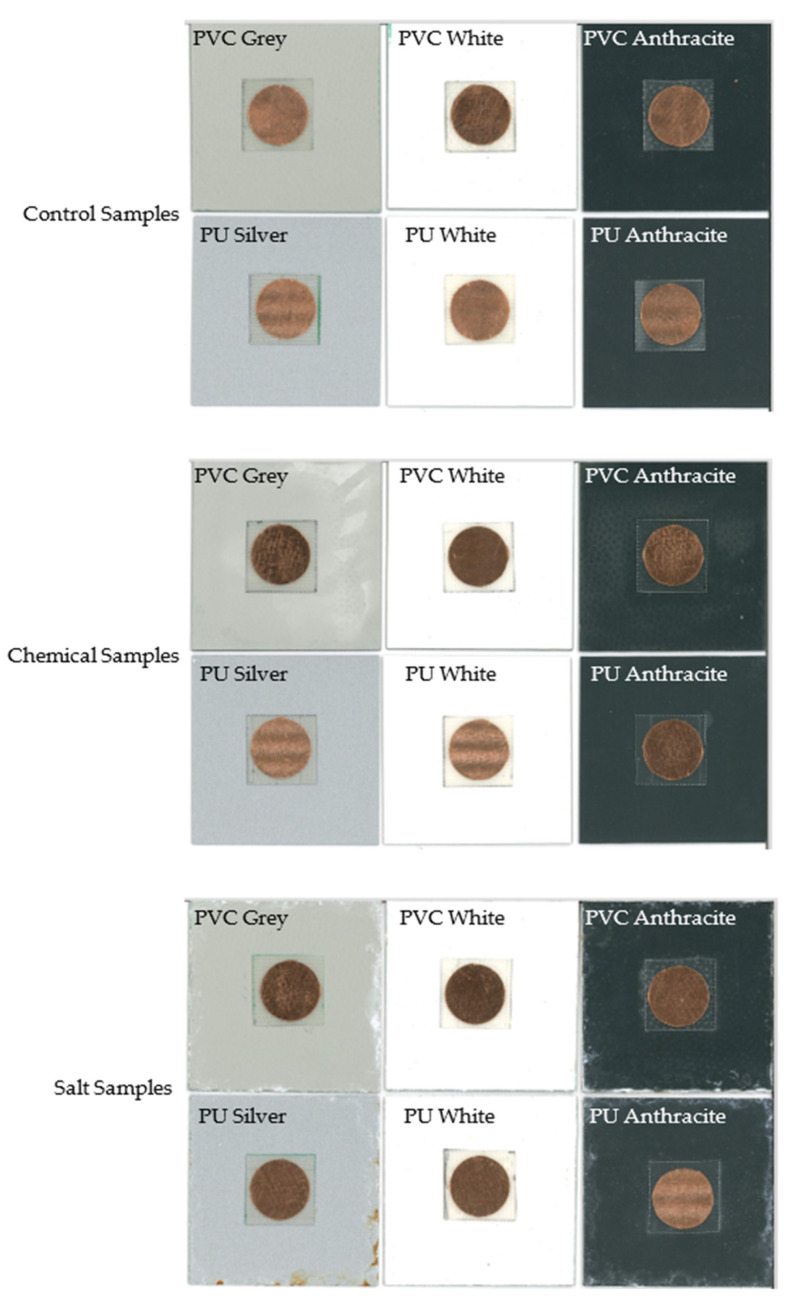
Sample appearance after no testing (control) (**top**), chemical (**middle**) and salt (**bottom**) exposure testing.

Following the initial testing of the chipless RFID system, samples were produced with different artificial aging techniques. The set of samples (Salt) was produced via 10 weeks of ASTM B117 salt fog/spray exposure in a 100% humidity (5% NaCl solution, pH 7), 35 °C environment. A second set of samples (Chemical) was exposed to 50 MEK (Methyl Ethyl Ketone) double rubs, a 30 min acetone soak, and a two-hour boil. This was done as a proof of the concept of the technique to detect changes in organic coatings caused by degradation, and hence the use of common accelerated weathering techniques. The samples after exposure are shown in Figure 17.

**Figure 18:**
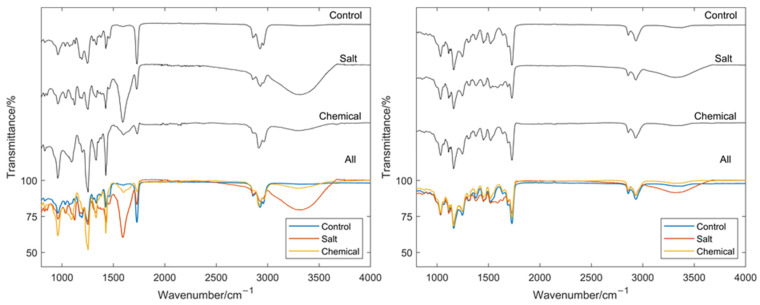
Resulting FTIR spectra for an example PVC sample (**left**) and example PU sample (**right**).

It can be seen that the chemical samples do not appear, visually, that different to the control samples except for slight discoloration on the PVC grey and anthracite samples. The salt exposed samples show a far greater visual level of degradation with significant blistering and corrosion at the cut edge. However, it should be noted that the sensor tag is only expected to detect changes in the coating directly underneath where it is placed and that the middle of the samples showed little visual change to the control samples. Hence, the sensor is still monitoring an area in which degradation of the coating has not occurred significantly enough for a visual inspection to determine. After exposure to the environments, the samples were also examined using FTIR. Examples of the FTIR results are shown for a PVC and PU sample in Figure 18.

**Figure 19:**
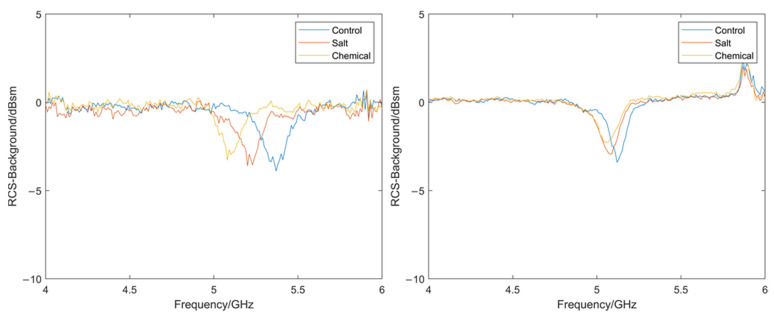
Measured RCS for a PVC coating system (**left**) and a PU coating system (**right**) samples exposed to salt and chemical degradation.

From these spectra, it is possible to see the effect the different treatments have had on the two paint systems. Perhaps most obvious is the significant emergence of the characteristic broad OH peak between approximately 3000 and 3500 cm^−1^ [42] in the samples exposed to the salt spray. This is indicative of moisture presence either in the coating itself or through the formation of micropores or blisters. While the chemically exposed samples do also perhaps show slight emergence of this peak, especially in the PVC samples, the effect is not as pronounced. However, from these results, it would be expected that the salt exposed samples would have a significantly different, higher, dielectric constant and that the chemically exposed samples may have a slightly higher dielectric constant. The respective obtained RCS results for a PVC and PU sample are shown in Figure 19.

**Figure 20:**
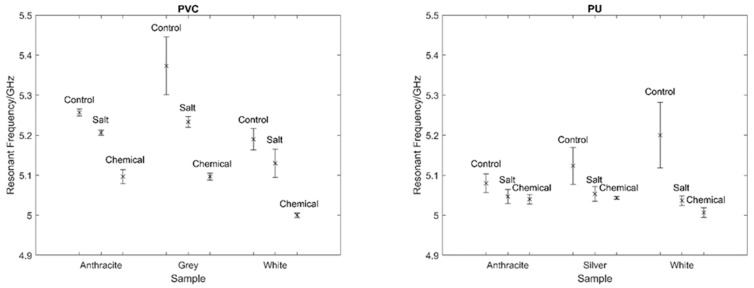
Measured resonant frequency for each PVC (**left**) and PU (**right**) sample tested and the associated standard error.

It can be observed that the salt and chemical treatment has caused the resonant frequency to shift to the left and that this shift is greater in magnitude for the PVC sample. This shift is indicative of the types of changes expected under these tests. A comparison of the resonant frequencies for all the tested samples exposed to the salt and chemical degradation can be seen in Figure 20 where the resonant frequency was measured using three repeats. All the samples tested show a similar trend to that of the example above, even with the variation in measurements.

As expected, the shift in resonant frequency as a result of coating changes for the PU samples is far more reduced than for that of the PVC samples. As mentioned previously, the additional layer of FR-4 used to increase the signal for both paint systems effectively reduces the magnitude of the relative shift in resonant frequency due to its unchanging dielectric properties. The influence of this effect is far greater in the PU sample, where the FR-4 thickness (0.2 mm) constitutes a far larger proportion of the total antenna dielectric; approximately 83% compared to 50% of the total PVC antenna dielectric. However, despite this, it is still possible to distinguish the different treatments through resonant peak identification.

## 4. Discussion

There is no doubt that the monitoring of organic coatings applied to steel cladding on buildings is a difficult proposition. The current common monitoring practices for buildings are often simple and subjective manual inspections, and generally, a ‘fix when failed’ approach is taken. This means that degradation is only detected when significant coating damage and/or corrosion has taken place, increasing the cost of rectification. Recent techniques developed for monitoring early-stage coating deterioration suffer from requirements to modify the coating layers or connect to the substrate making results less representative of the bulk and more complex to carry out in-situ.

While not a perfect solution, the novel method developed shows considerable promise for the detection of pre-failure degradation in coated steels, which could be carried out quickly and reliably at low cost. It has shown its ability to detect both water uptake by the coating/coating–metal interface and degradation of the polymer itself before a significant visual change in the sample area has occurred. The degree of degradation is expected to be semi-quantitatively assessed by considering the size of the resonant frequency shift between the virgin sample and exposed sample with a greater degree of degradation producing a larger shift.

However, to further investigate and develop the technique, a number of additional studies are required. The sensitivity of the method needs to be established such that the change in resonant frequency can be more linked to the degree of degradation. For example, coating material integrity is often affected by UV, and this would be expected to provide a change in the dielectric properties of the coating. Typically, the change in structure that occurs when a coating is subjected to UV arises from the breakdown of the longer polymer chains, pigment bleaching, and cracking of the coating [41]. The precise impact of this and other failure modes on the dielectric properties of the coating should be addressed. This requires multiple coated substrate samples of varied levels of degradation, through multiple degradation mechanisms to be measured and analyzed.

Furthermore, the universality of the resonance value for multiple coating systems needs to be established. The absolute resonance is a function of many coating and substrate parameters. Hence, the coating systems of interest must first be tested in advance to determine the specific resonant frequency for the given coating thickness, pigmentation, polymer system and application involved. Thus, this technique would be used to monitor any resonance shift from this specific virgin sample datum, which will need to be considered as the prime indicator of coating degradation/substrate corrosion. It is therefore a relative, not an absolute measurement technique. Given the background noise observed within the laboratory, signal processing techniques may need to be refined in order to clearly identify the peaks in a consistent manner. 

Finally, if the laboratory findings are positive then a simplified robust piece of hardware needs to be designed which can be used in the field. All of the hardware electronics for such a device exists and is relatively low cost, but it would need to be ruggedized for use in practice.

It is envisaged that this technique could be deployed via miniaturization into a portable handheld device, which is used to interrogate a tag that can be placed and then removed during routine inspections. The device would ensure that the interrogating antennas are fixed at a required angle and distance for measurement and a ‘place and remove’ testing procedure would ensure tarnishing of the tags does not impact the results and would reduce the aesthetic implications of constantly mounted tags. The resonant frequency measured could then be compared to the baseline initially measured resonant frequency and the shift related to the condition of the coating system. As each tag is only influenced by the coating directly beneath it several locations would have to be tested. However, this would allow a determination of the general condition of the coating across the building. Where the technique suggests early-stage coating degradation is occurring more rapidly than expected, maintenance and/or repair of the coating, via overpainting for example, would reduce the likelihood of degradation continuing to the point where replacement is required.

## 5. Conclusions

This paper has introduced a new non-contact, non-destructive method for the monitoring of organically coated steel. The design of a CMPA was achieved via simulation and, by considering shifts in the resonant frequency of the CMPA, was shown to provide information as to the condition of an organic coating, even when the organic coating is relatively thin. It was observed that as a coating underwent simulated ageing, the expected response would be a decrease in the resonant frequency due to an increased dielectric constant and reduced thickness. For thinner coatings, the use of an additional piece of FR-4 substrate allowed a more substantial response, although it reduced the sensitivity of the method. Experimental results showed that background noise was the largest cause for concern, and hence resonant peaks could only be distinguished accurately if additional substrate thickness was used. Background removal via the measurement of the sample background RCS was also crucial to allow further ease of peak detection. However, the technique did respond as expected in the simulations when determining the resonant frequency of samples exposed to accelerated weathering conditions. These samples showed a clear decrease in resonant frequency as a result of water uptake and polymer degradation. This method shows promise as a rapid way to determine coating condition non-destructively and without contact with the product being tested.

## Figures and Tables

**Table 1:** Simulation parameters.

Component	Symbol	Dimension/mm
Antenna Radius	r	10
Antenna Thickness	t	0.035
Dielectric Thickness	pt	0.211 (PVC) 0.040 (PU)
Zinc Thickness	zt	0.04
Metal Thickness	mt	0.6
Dielectric Constant (PVC & PU)	e	3.5
Sample Height/Width	m	60
Substrate Thickness	st	0.00 (NS) 0.20 (S)
Substrate Height/Width	s	0.00 (NS) 22.0 (S)

**Table 2:** Simulation material properties.

Component	Value/Properties	Reference
Steel Conductivity (σ_S_)	6.99 × 10^6^ S/m	[32,33]
Zinc Conductivity (σ_z_)	1.69 × 10^7^ S/m	[32,33]
Fr-4 Dielectric Constant (Ɛ_r_)	4.3	[33]
Fr-4 Loss Tangent (δ)	0.025	[33]

**Table 3:** Results of initial simulations.

System	Resonant Frequency/GHz	RCS at Resonant Frequency/dBsm	RCS Change/dBsm
PVC-NS	4.556	−17.41	3.48
PU-NS	4.672	−13.87	0.03
PVC-S	4.380	−17.43	3.33
PU-S	4.244	−15.17	1.02

**Table 4:** Calculated optimum range of dielectric height.

System	Resonant Frequency/GHz	System h/mm	h Min/mm	h Max/mm
PVC-NS	4.556	0.211	0.20	3.29
PU-NS	4.672	0.040	0.19	3.21
PVC-S	4.380	0.411	0.21	3.42
PU-S	4.244	0.240	0.21	3.53

**Table 5:** Parameters used for each aging severity level.

Aging Severity	Dielectric Constant	Defect Diameter/mm	Paint Thickness/mm
1	3.500	0.00	0.211 (PVC) 0.040 (PU)
2	3.525	0.01	0.210 (PVC) 0.039 (PU)
3	3.550	0.02	0.209 (PVC) 0.038 (PU)
4	3.575	0.03	0.208 (PVC) 0.037 (PU)
5	3.600	0.04	0.207 (PVC) 0.036 (PU)
6	3.625	0.05	0.206 (PVC) 0.035 (PU)
7	3.650	0.06	0.205 (PVC) 0.034 (PU)
8	3.675	0.07	0.204 (PVC) 0.033 (PU)

## Data Availability

All data supporting this study are provided in full in the ‘Results’ section of this paper.
